# Expression of interferon-induced antiviral genes is delayed in a STAT1 knockout mouse model of Crimean-Congo hemorrhagic fever

**DOI:** 10.1186/1743-422X-9-122

**Published:** 2012-06-19

**Authors:** Gavin C Bowick, Adriana M Airo, Dennis A Bente

**Affiliations:** 1 Department of Microbiology & Immunology, Center for Biodefense and Emerging Infectious Diseases, Sealy Center for Vaccine Development, Institute for Human Infections & Immunity, Galveston, TX, USA; 2 Center for Tropical Diseases, University of Texas Medical Branch, Galveston, TX, USA; 3 Special Pathogens Program, National Microbiology Laboratory, Public Health Agency of Canada, Winnipeg, MB, Canada; 4 Department of Microbiology & Immunology, 301 University Boulevard, Galveston, TX, 77555-0610, USA

**Keywords:** Crimean Congo hemorrhagic fever, Interferon, Animal model, Signaling, STAT1

## Abstract

**Background:**

Crimean Congo hemorrhagic fever (CCHF) is a tick-borne hemorrhagic zoonosis associated with high mortality. Pathogenesis studies and the development of vaccines and antivirals against CCHF have been severely hampered by the lack of suitable animal model. We recently developed and characterized a mature mouse model for CCHF using mice carrying STAT1 knockout (KO).

**Findings:**

Given the importance of interferons in controlling viral infections, we investigated the expression of interferon pathway-associated genes in KO and wild-type (WT) mice challenged with CCHF virus. We expected that the absence of the STAT1 protein would result in minimal expression of IFN-related genes. Surprisingly, the KO mice showed high levels of IFN-stimulated gene expression, beginning on day 2 post-infection, while in WT mice challenged with virus the same genes were expressed at similar levels on day 1.

**Conclusions:**

We conclude that CCHF virus induces similar type I IFN responses in STAT1 KO and WT mice, but the delayed response in the KO mice permits rapid viral dissemination and fatal illness.

## Findings

*Crimean-Congo hemorrhagic fever virus* (CCHFV) is a *Nairovirus* of the *Bunyaviridae* and is the etiological agent of Crimean-Congo hemorrhagic fever (CCHF). The virus is associated with mortality rates from 5 to 70% [1]. The pathogenesis of the disease is largely not understood due the lack of a suitable animal model. We recently described the establishment of an adult mouse model for CCHF using STAT1 knockout (KO) mice. STAT1 KO mice are highly susceptible to the virus and the mouse model exhibits key features seen in human cases of CCHF. Mice lacking interferon responses have been increasingly used as models for other hemorrhagic fever viruses, including the families flaviviridae, filoviridae, arenaviridae, and bunyaviridae [2-8].

Using the STAT1 KO model, we noted the production of high levels of interferon α and β in plasma of infected mice [9]. We hypothesized that, despite the lack of STAT1, this interferon may lead to activation of certain interferon-regulated genes, possibly by signaling through alternative pathways. We used interferon α/β response PCR arrays (SA Biosciences, Frederick, MD) to investigate gene expression changes in the spleen and the liver of KO and wild-type (WT) mice infected with CCHFV at 1, 2 and 3 days post-infection. Experiments were conducted as previously described [9]. Briefly, KO and WT mice were challenged intraperitoneally with 100 PFU of CCHFV strain IbAr 10200. Livers and spleens were harvested on day 1, 2 and 3 post-infection. Gene expression of infected KO and WT mice was compared to mock infected KO and WT mice. Liver and spleen were chosen as they were the tissues that showed histopathological changes and had the highest viral titers [9]. All animal studies were performed in accordance with institutional animal care and use protocols. An overview of all data is provided in Additional file 1: Tables S1 and Additional file 2: Tables S2. We noted modest induction of STAT1 in the KO mice; we attribute this to the incomplete knockout of all exons of the STAT1 gene which leads to production of a non-functional STAT1 protein [10].

**Figure 1:**
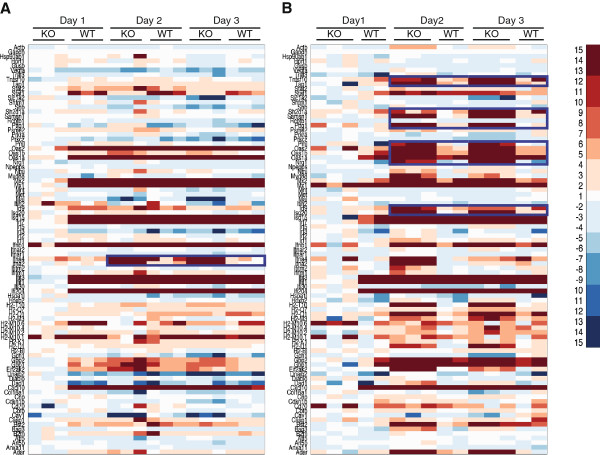
** Overview of gene expression changes following CCHFV infection in the spleen (A) and liver (B).** STAT1 KO and wild-type mice were infected with CCHFV and RNA harvested at 1, 2, and 3 days post-infection for expression analysis by real-time RT-PCR. Data were normalized to GAPDH levels and represented as heatmaps. Upregulated genes are shown in red, downregulated genes are shown in blue. Fold changes beyond 15 and −15 remain at the same shade as fold changes of 15 and −15.

Heat maps were plotted using MATLAB (The Mathworks, Natick, MA). Figure 1A shows that semi-quantitative expression profiles of the majority of genes are consistent between all groups, with two exceptions. The first is the lack of gene upregulation by the KO mice at 1 day post-infection. The second is the lack of significant induction if IFN-α2 and IFN-α4 by the WT animals (box). Gene expression in the liver showed a number of differences in expression pattern compared to that observed in the spleen (Figure 1B). Again, the KO mice did not upregulate many genes at 1 day post infection. However, at days 2 and 3 post-infection, KO mice showed increased numbers of upregulated genes compared to WT mice (boxed).To investigate the upstream transcription factors which may be involved in controlling the observed pattern of gene expression, we used PSCAN [11], which we have previously used to analyze a number of high-throughput hemorrhagic fever datasets [12]. As expected, the analysis revealed IRF1 and IRF2 as key transcription factors involved in the control of these responses. However, at 1 day post-infection, Egr1 and En1 were identified as enriched transcription factor binding sites in genes downregulated in WT mice, but not KO mice. At 3 days post-infection, NF-κB was implicated in transcriptional upregulation in addition to IRFs 1 and 2, possibly due to the increased levels of TNF-α observed at this time [9].

**Figure 2:**
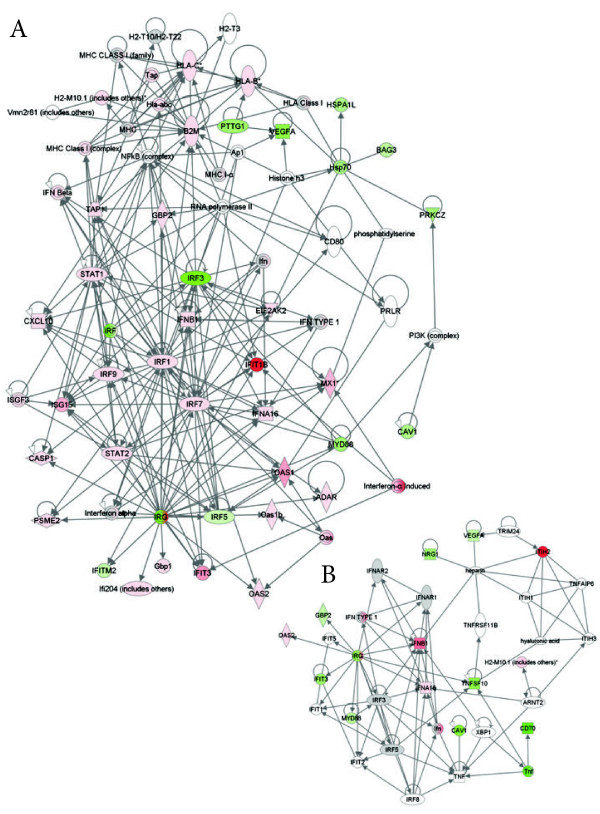
** Signaling networks induced in the spleen at 1 day post infection in wild-type mice (A) and STAT1 KO mice (B).** Gene lists of significantly (fold change <2, p < 0.05) differentially expressed transcripts were used to construct functional signaling networks on the basis of know interactions using the Ingenuity Pathway Analysis software. Only direct interactions were used to construct networks and only pathways with scores >5 (*p* < 10^-5^) were included in the merged networks.

We sought to place these findings in a functional immune signaling context. We used Ingenuity Pathway Analysis (Ingenuity Systems, Redwood City, CA) to construct signaling networks on the basis of known interactions. We have previously used this software to define cell signaling changes associated with the arenavirus, *Pichindé virus*[13-15]. The network following infection of the WT mouse (Figure 2A) was more complex than that seen following infection of the KO mice (Figure 2B). Both networks were built around interferon signaling, however, the network induced in WT mice included other key transcription factors including c-Myc, CREBBP (cAMP-responsive element binding, binding protein), p53 and NF-κB. Taken together, these analyses suggest that a more coordinated cellular response to infection, integrating multiple signaling pathways, is produced early in the WT mouse and is required for protection from lethal disease.

We next characterized the significance of specific cellular functions and pathways during infection (Additional file 3: Figure S1). At 1 day post infection, both groups of mice showed similar significance of antigen presentation and immune cell trafficking in the spleen. However, WT mice showed a greater transcriptional response of genes involved in the inflammatory response, antimicrobial response, gene expression, post-translational modification and protein folding. Analysis of gene expression associated with canonical pathways revealed increased numbers of differentially expressed genes associated with the roles of pattern recognition receptors (PRRs), interferon signaling, interferon response factor activation by PRRs, dendritic cell maturation and PKR induction of the antiviral response in the WT mice at 1 day post-infection. However, by 2 days post-infection, there were no striking differences between the pathway significance profiles between the two groups, suggesting that, by 2 days post-infection, KO mice are responding qualitatively similarly to WT mice.

**Figure 3:**
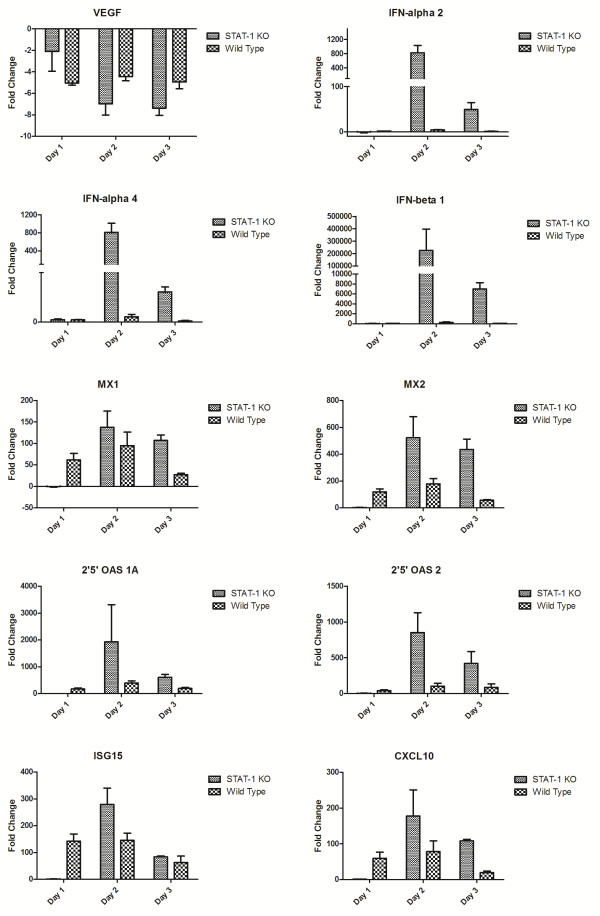
** Expression levels of transcripts in the spleens of STAT1 KO or wild-type mice infected with CCHFV at 1, 2 and 3 days post-infection (*n*** **= 3 mice per group).**

We next looked at the magnitude and kinetics of expression profiles of individual genes in the spleen (Figure 3). As seen in the heatmaps, the majority of genes showing significant (*p* < 0.05) expression changes showed a lack of induction in the KO mice at 1 day post-infection. Some genes, such as VEGF, showed a similar pattern of expression between both groups. IFN-α2, α4 and β1 were significantly upregulated in KO mice. Given that KO mice should be defective in signaling downstream of the interferon receptor, it had been predicted that the inability of IFN to induce antiviral gene expression was responsible for pathogenesis. However, an interesting finding is that, by 2 days post-infection, many of the genes associated with upregulation by interferons, such as Mx1, Mx2 and 2’5’ oligoadenylate synthase (OAS) demonstrate high levels of expression.

**Figure 4:**
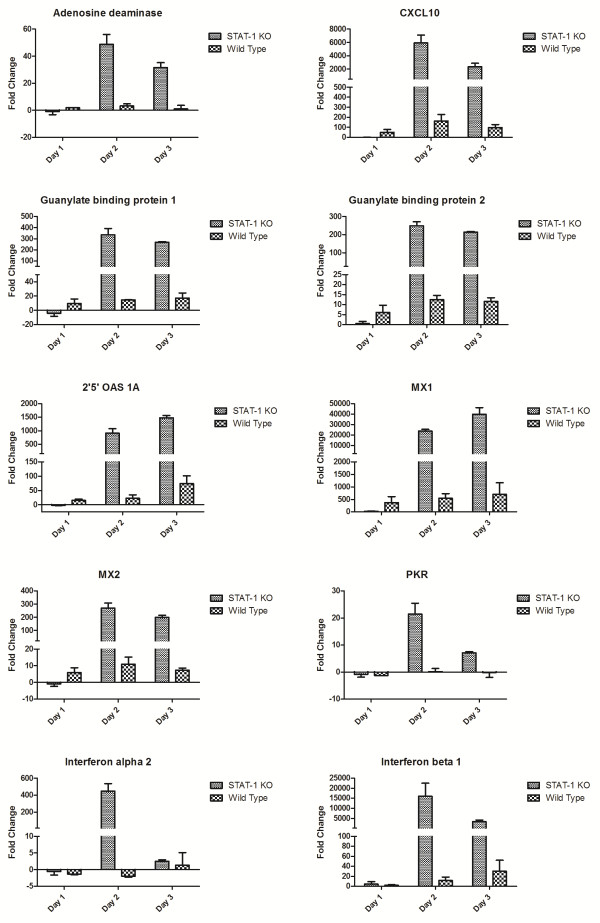
** Expression levels of transcripts in the livers of STAT1 KO or wild-type mice infected with CCHFV at 1, 2 and 3 days post-infection (*n*** **= 3 mice per group; 2 mice per group for wild-type mice).**

A similar pattern of expression was also observed in the liver (Figure 4), although the levels of IFN target genes were strikingly higher in KO mice than WT mice for many genes, with fold changes of genes such as adenosine deaminase, CXCL10, guanylate-binding proteins (Gbp)-1 and −2, 2’5’ OAS and the Mx proteins being an order of magnitude higher in KO mice by day 2 post-infection. Taken together, these data suggest that high levels of induction of antiviral genes, such as PKR, Mx1 and 2, Gbp-1 and −2 and 2’5’OAS are insufficient to protect against lethal CCHFV-induced disease in this model.

In this report, we have shown that infection of STAT KO mice with CCHFV leads to induction of many interferon-induced genes associated with the antiviral response, but that this response is delayed compared to infection of WT mice. STAT1 KO mice have been used in a number of studies using a range of pathogens [16-22]. Similar to what we demonstrated here, some of these studies reported STAT1-independent IFN pathway gene expression patterns. A recent study demonstrated that much of the STAT1-independent activity is dependent on STAT2, since mice lacking both STAT1 and STAT2 show susceptibility to dengue virus [23]. Our report is consistent with the observations of STAT1-independent type-I IFN signaling described in this study, with STAT1 being required for an early activation of type-I IFN production and antiviral gene expression. Consistent with our mouse model, several studies showed an increased replication in target tissues most likely due to lack of antiviral state of cells allowing the virus to replicate in more cells compared to the wildtype [17,18,22]. Interestingly, we also demonstrated residual STAT1 activity in this study, a phenomenon also described by another group [24]. At this point, it is unclear whether the IFN-dependent gene expression seen in this study arose from STAT1-independent pathways, might have resulted, at least in part, from a small amount of residual STAT1 activity, or if they are induced by another mechanism, e.g., the virus itself or by other cytokines. Further studies in our laboratory will focus on elucidating this mechanism.

Many of the findings described are consistent with our previous report [9] and provide a basis for these observations. Interestingly, in human cells, the type-I interferon response and the MxA protein in particular have been shown to inhibit the replication of CCHFV [25,26]. Others have demonstrated with Dugbe virus, closely related to CCHFV, that certain IFN genes such as PKR and MxA are not sufficient for protection in a mouse model [27]. Our data shows that induction of these genes is insufficient for protection against CCHFV. This may reveal species-specific differences, *in vitro* versus *in vivo* differences, or could be further evidence that the delay in induction of antiviral genes is critical.

In summary, we have further characterized the STAT1 KO mouse model for CCHFV infection. Given our finding of significant antiviral gene expression, we postulate that, as discussed in our previous characterization of the model, that, while the IFN response is critical in the control of CCHFV, it appears the kinetics of the response, combined with the activation of multiple pathways, is the most important factor in determining the outcome of infection.

## Competing interests

The funders had no role in study design, data collection and analysis, decision to publish, or preparation of the manuscript. The authors have no conflicts of interest to disclose.

## Authors’ contributions

DAB conceived the study, DAB and AMA performed experiments, GCB performed data and network analysis, GCB and DAB wrote the manuscript. All authors read and approved the final manuscript.

## Supplementary Material

Additional file 1 **Table S1.** Complete list of fold changes in an Excel file. Click here for file

Additional file 2 **Table S2.** Summary list of up and downregulated genes shown as common name and GenBank accession number in an Excel file. Click here for file

Additional file 3 **Figure S1.** Functional and pathway significance. The Ingenuity Pathway Analysis application was used to show the association of differentially expressed genes with canonical functions or pathways in the spleen. The significance level does not indicate a degree of functional activity, but is a statistical measure of the number of differentially expressed genes associated with the function or pathway. The analysis shows functional associations at 1 day post-infection (A) and canonical pathway involvement at 1 and 2 days post-infection (B, C). Click here for file
